# Irreducible Posterior Fracture Dislocation of the Shoulder: A Case Report

**DOI:** 10.7759/cureus.33819

**Published:** 2023-01-16

**Authors:** Otmane Sammouni, Saber Zari, Najib Abdeljaouad, Hicham Yacoubi

**Affiliations:** 1 Traumatology-Orthopedics Department, Mohammed VI University Hospital, Faculty of Medicine and Pharmacy of Oujda, Mohamed I University, Oujda, MAR

**Keywords:** surgical treatment, rare, complex, shoulder, posterior fracture-dislocation

## Abstract

Posterior shoulder fracture-dislocation is a rare traumatic entity. Early diagnosis results in the adequate treatment of these lesions and prevents serious complications, such as avascular necrosis.

Several therapeutic options have been proposed depending on the size of the humeral defect, duration of the dislocation, age of the patient, associated comorbidities, and functional requirements. For this kind of injury, early open anatomical reduction and stable internal fixation remain the optimum treatment option. Arthroplasty is often the last resort for active young patients, particularly in cases of avascular necrosis, humeral head complex fracture, or undiagnosed posterior shoulder dislocation.

Here, we report a case of a complex posterior shoulder fracture-dislocation in a young man, which was treated surgically with open reduction and locked plate osteosynthesis using a deltopectoral approach.

## Introduction

Posterior shoulder dislocation represents fewer than 5% of all shoulder dislocations. According to Neer and Foster, when associated with a fracture, it is much more uncommon [1,2]. PDS can be complicated by a fracture of the anatomical neck, known as a complex posterior shoulder fracture-dislocation. It is most commonly observed in highly kinetic trauma [3] and seizures and electrocution, in which the mechanism is a forced flexion associated with adduction and internal rotation of the shoulder [4]. Early diagnosis results in the adequate treatment of these lesions and prevents serious complications, such as avascular necrosis [5].

Several therapeutic options have been suggested for these lesions, ranging from non-surgical methods to surgical repair or prosthetic replacement; however, optimal treatment management remains a subject of controversy [6-8].

Here, we report a case of a complex posterior shoulder fracture-dislocation in a young man, which was treated surgically with open reduction and locked plate osteosynthesis using a deltopectoral approach.

## Case presentation

**Figure 1:**
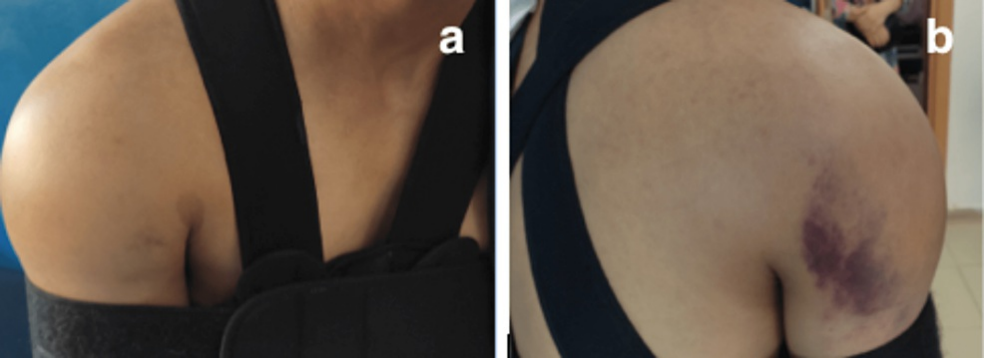
Clinical aspect of the patient's right shoulder after the trauma showing edema and ecchymosis posteriorly.

A 34-year-old man with no history of pathology fell from a scaffold (estimated at 6 m high). The clinical examination revealed a painful, edematous right shoulder with ecchymosis on the posterior surface, The vascular-nervous examination was normal (Figure 1).

**Figure 2:**
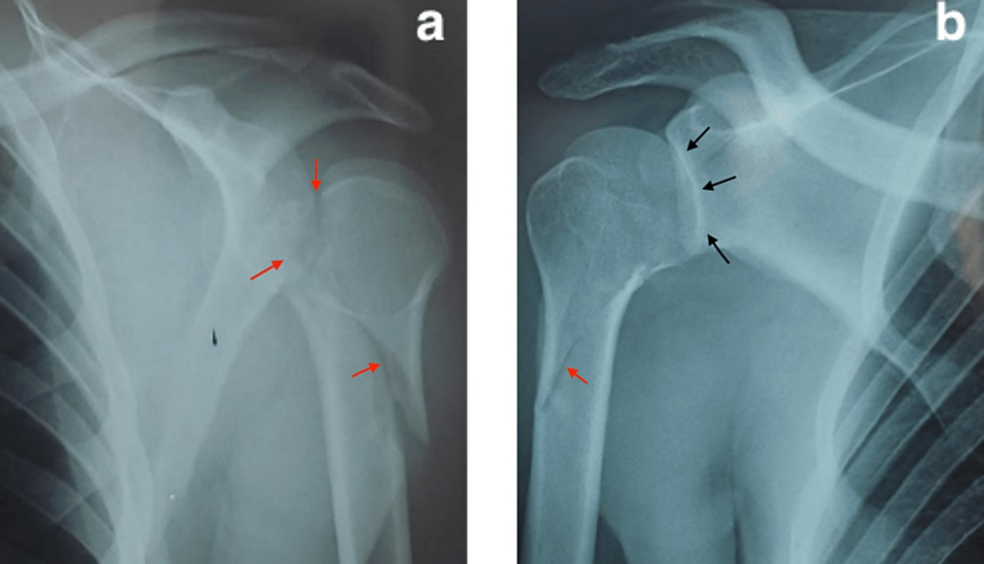
X-rays of the right shoulder after the trauma showing a fracture (red arrow) of the right proximal humerus, with a posterior dislocation (black arrow) of the humeral head.

**Figure 3:**
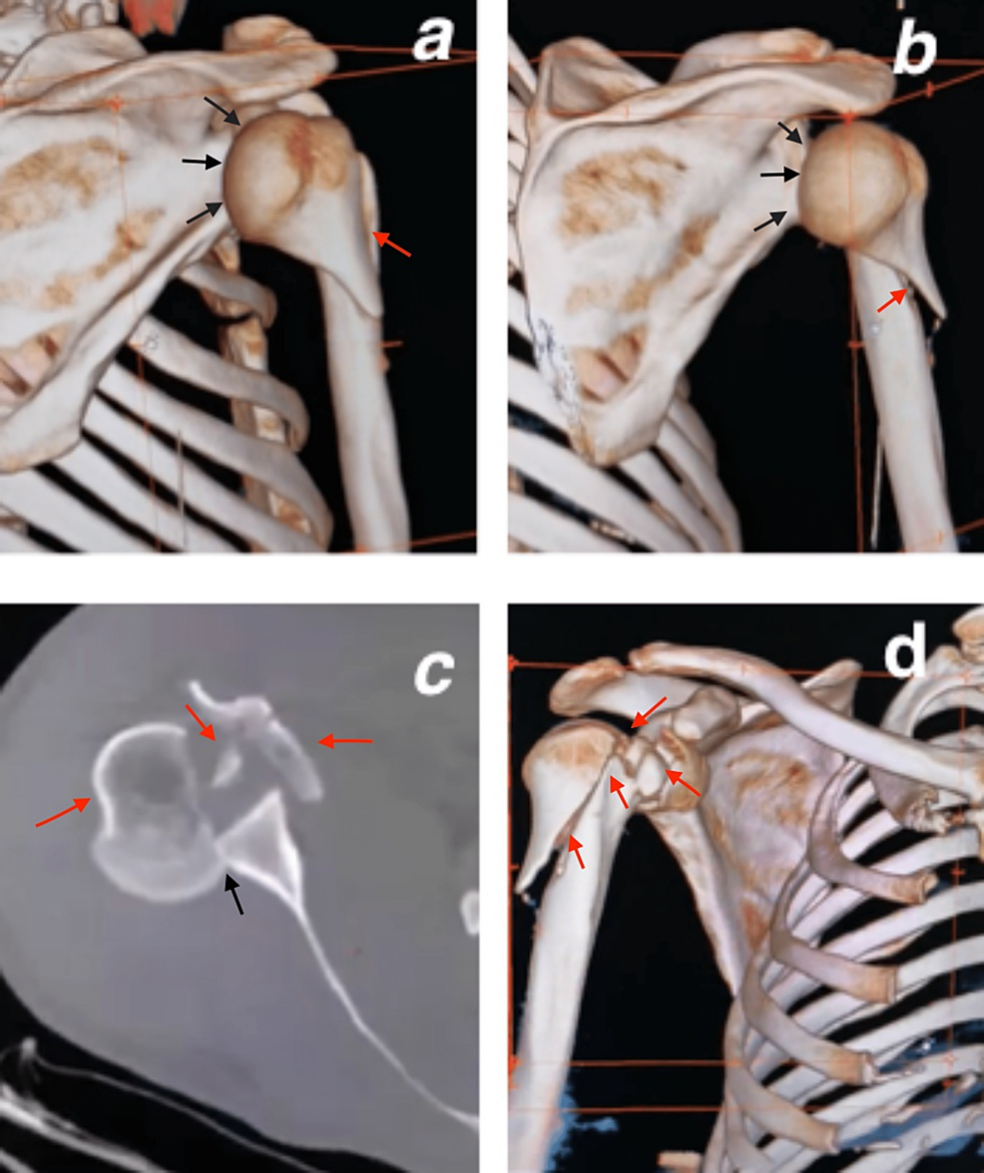
CT scan of the right shoulder after the trauma shows the three-dimensional reconstruction slices (a-d), axial slice (c), and a complex three-part posterior fracture (red arrow) with dislocation (black arrow) of the right shoulder.

The radiological assessment using standard radiographs (Figure 2) and CT scan (Figure 3) of the right shoulder showed a posterior shoulder dislocation with a complex three-part fracture of the humerus proximal extremity, as classified by Neer and Foster [2], with failure of the closed reduction, which was performed under sedation.

**Figure 4:**
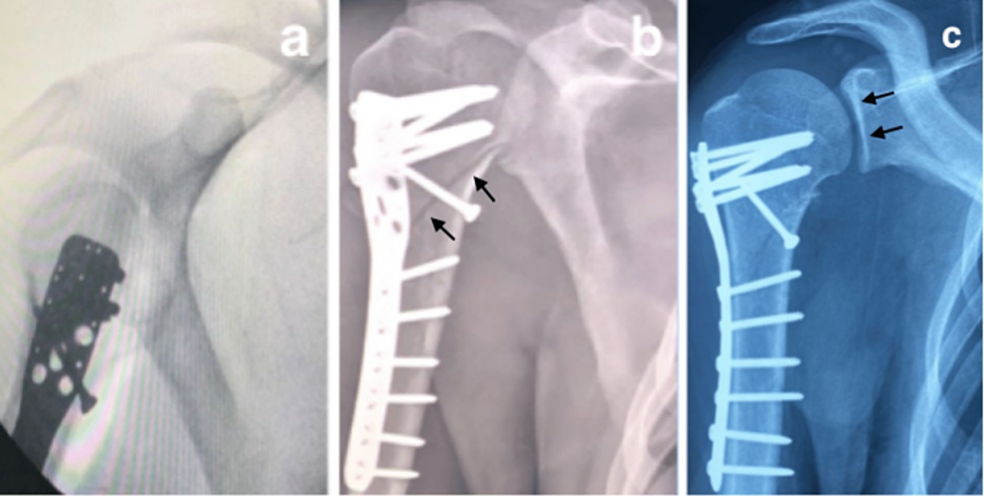
Intraoperative (a), postoperative (b), and three-week radiographs showing good reduction of the posterior shoulder fracture-dislocation (black arrow).

Surgical management was performed under general anesthesia in a beach-chair position, using the deltopectoral approach. The first surgical step consisted of disengaging the proximal portion of the humerus blocked posteriorly. After debridement and adequate exposure, the reduction was obtained by traction in the axis of the upper limb, with disimpaction of the humeral head using a Kocher forceps from the belly of the deltoid muscle, we maintained fracture reduction provisionally with forceps. Then, we applied a direct screw perpendicular to the fracture line to approve the reduction. The second surgical step consisted of placing a locked plate just below the greater tuberosity and slightly behind the bicipital groove. We passed heavy sutures through the subscapular and supraspinatus tendons and through the plate to reduce the fracture fragments of the lesser tuberosity. Intraoperative and postoperative radiographs showed a good reduction of the fracture and the posterior shoulder dislocation (Figures 4a, 4b). The rehabilitation protocol was started on the second postoperative day, with pendular movements of the shoulder with flexion and extension of the elbow helped by the contralateral hand. In the third week, we started active movements (Figure 4c).

**Figure 5:**
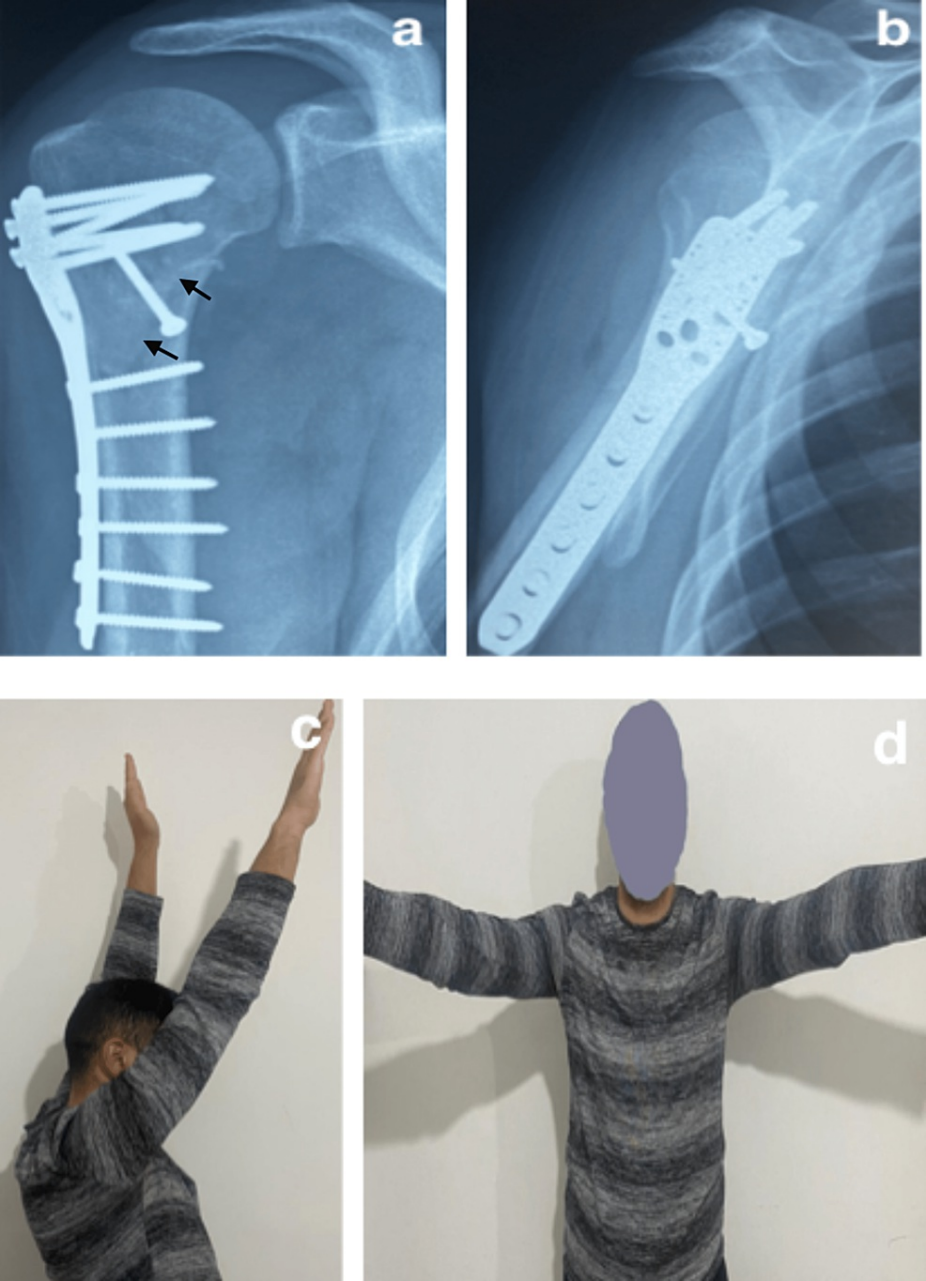
(a, b) Radiographs at six weeks showing consolidation of the posterior fracture-dislocation (black arrow), (c, d) with satisfactory shoulder function at six months.

The patient’s postoperative clinical and radiological evolution was closely monitored, and the results were favorable. Radiography of the shoulder at six weeks had objectified a complete consolidation (Figures 5a, 5b). At the last visit, the patient was very satisfied (Figures 5c, 5d) with a Constant score of 85/100 [9].

## Discussion

The posterior dislocation fracture is a rare lesion. Despite its rarity in daily clinical practice, it remains an important diagnosis because it is observed, especially in young active patients, and often goes unnoticed [10]. It has been reported to escape diagnosis at the first examination in most cases, which leads to complications, such as stiffness, persistent pain, and thus the inability to perform the movements of daily life [11-13], not to mention the major risk of avascular necrosis of the humeral head if the reduction is not performed within 48 hours of the injury [5]. An early and thorough diagnosis associated with a complete radiological assessment is the key element for well-adapted management [14].

In the case of a suspected dislocation fracture of the shoulder, the authors recommend a series of three views, namely, anterior-posterior, scapular Y, and axillary. Nowadays, CT has become a valuable examination tool for confirming these types of lesions before any attempt at closed reduction [6,12] and helps in planning the surgical procedure [1,15].

The surgical management of posterior shoulder dislocation with fractures is a controversial issue as there are various therapeutic means that vary according to the age of the lesion and the percentage of defects of the humeral head, along with the patient’s associated factors (such as age, history) [16]. Early open anatomical reduction and stable internal fixation remain the optimum treatment option for young and active patients, arthroplasty is often the last resort, particularly in cases of avascular necrosis, humeral head complex fracture, or undiagnosed posterior shoulder dislocation. Several approaches have been described by the authors for this type of lesion, with the deltopectoral approach being the most commonly used, especially to expose the anterior glenohumeral joint [1], which we used in our patient. The posterior approach is frequently used in dislocations with a significant defect of the humeral head [17]. It is known widely that the crucial factor in avoiding capsular contracture and the subsequent shoulder stiffness that follows the surgical treatment of a proximal humerus fracture is prompt initiation of motion and rehabilitation.

In our case, as the patient was young and active, we adopted an early open anatomical reduction and internal fixation while ensuring a good and immediate rehabilitation which explains the favorable outcomes.

## Conclusions

Posterior shoulder fracture-dislocations are rare injuries. The key components of well-adapted management are an early physical examination and a complete radiological workup. The optimum treatment option for this kind of fracture in young and active patients remains open reduction and stable internal fixation.

## References

[1] Kowalsky MS, Levine WN (2008). Traumatic posterior glenohumeral dislocation: classification, pathoanatomy, diagnosis, and treatment. Orthop Clin North Am.

[2] Neer CS 2nd, Foster CR (1980). Inferior capsular shift for involuntary inferior and multidirectional instability of the shoulder. A preliminary report. J Bone Joint Surg Am.

[3] Robinson CM, Akhtar A, Mitchell M, Beavis C (2007). Complex posterior fracture-dislocation of the shoulder. Epidemiology, injury patterns, and results of operative treatment. J Bone Joint Surg Am.

[4] Saltzman BM, Erickson BJ, Harris JD, Gupta AK, Mighell M, Romeo AA (2016). Fibular strut graft augmentation for open reduction and internal fixation of proximal humerus fractures: a systematic review and the authors' preferred surgical technique. Orthop J Sports Med.

[5] Schnetzke M, Bockmeyer J, Loew M, Studier-Fischer S, Grützner PA, Guehring T (2018). Rate of avascular necrosis after fracture dislocations of the proximal humerus: timing of surgery. Obere Extrem.

[6] Rouleau DM, Hebert-Davies J (2012). Incidence of associated injury in posterior shoulder dislocation: systematic review of the literature. J Orthop Trauma.

[7] Sheehan SE, Gaviola G, Gordon R, Sacks A, Shi LL, Smith SE (2013). Traumatic shoulder injuries: a force mechanism analysis-glenohumeral dislocation and instability. AJR Am J Roentgenol.

[8] Schliemann B, Muder D, Gessmann J, Schildhauer TA, Seybold D (2011). Locked posterior shoulder dislocation: treatment options and clinical outcomes. Arch Orthop Trauma Surg.

[9] Constant CR, Murley AH (1987). A clinical method of functional assessment of the shoulder. Clin Orthop Relat Res.

[10] Rowe CR, Zarins B (1982). Chronic unreduced dislocations of the shoulder. J Bone Joint Surg Am.

[11] Hawkins RJ, Neer CS 2nd, Pianta RM, Mendoza FX (1987). Locked posterior dislocation of the shoulder. J Bone Joint Surg Am.

[12] Castagna A, Delle Rose G, Borroni M, Markopoulos N, Conti M, Maradei L, Garofalo R (2009). Modified MacLaughlin procedure in the treatment of neglected posterior dislocation of the shoulder. Chir Organi Mov.

[13] Bock P, Kluger R, Hintermann B (2007). Anatomical reconstruction for reverse Hill-Sachs lesions after posterior locked shoulder dislocation fracture: a case series of six patients. Arch Orthop Trauma Surg.

[14] Paparoidamis G, Iliopoulos E, Narvani AA, Levy O, Tsiridis E, Polyzois I (2021). Posterior shoulder fracture-dislocation: a systematic review of the literature and current aspects of management. Chin J Traumatol.

[15] Gosens T, Poels PJ, Rondhuis JJ (2000). Posterior dislocation fractures of the shoulder in seizure disorders--two case reports and a review of literature. Seizure.

[16] Cicak N (2004). Posterior dislocation of the shoulder. J Bone Joint Surg Br.

[17] Karachalios T, Bargiotas K, Papachristos A, Malizos KN (2005). Reconstruction of a neglected posterior dislocation of the shoulder through a limited posterior deltoid-splitting approach. A case report. J Bone Joint Surg Am.

